# Optimising Wastewater Treatment: *Acinetobacter* sp. IrC1 as a potential multi-resistant bacterium for copper accumulation and dyes decolourisation

**DOI:** 10.21315/tlsr2023.34.3.3

**Published:** 2023-09-30

**Authors:** Wahyu Irawati, Triwibowo Yuwono, Reinhard Pinontoan, Valentine Lindarto

**Affiliations:** 1 Department of Biology Education, Universitas Pelita Harapan, Jalan M.H. Thamrin Boulevard No.1100, Kelapa Dua, Tangerang Regency, Banten 15811, Indonesia; 2 Department of Agricultural Microbiology, Universitas Gadjah Mada, Bulaksumur, Caturtunggal, Kec. Depok, Kabupaten Sleman 55281 Yogyakarta, Indonesia; 3 Department of Biology, Universitas Pelita Harapan, Jalan M.H. Thamrin Boulevard No.1100, Kelapa Dua, Tangerang Regency, Banten 15811,Tangerang, Indonesia; 4 Department of Natural Sciences, Sekolah Menengah Atas Dian Harapan Lippo Village, Tangerang, Indonesia

**Keywords:** Accumulation, Decolourisation, Copper, Dyes, Multi-Resistant

## Abstract

Improper disposal of waste containing copper and dye is an environmental issue that must be resolved immediately due to its harmful, non-degradable and toxic properties. Bioremediation efficiency can improve by cultivating copper and dye multi-resistant bacteria to remove various pollutant types simultaneously. This study aims at establishing the multi-resistance of *Acinetobacter* sp. IrC1 to copper and dyes. The effects of copper concentration on growth were determined using a spectrophotometer, while accumulation was analysed using an atomic absorption spectrophotometer. Bacteria-mediated dye decolourisation dyes were observed based on clear zone formation around bacterial colonies, while decolourisation percentage was calculated using a spectrophotometer. Results demonstrate that *Acinetobacter* sp. IrC1 resisted up to 8 mM CuSO_4_ and accumulated up to 292.93 mg/g dry weight of copper cells. *Acinetobacter* sp. IrC1 isolates were also resistant to 500 ppm Methylene Blue, Malachite Green, Congo Red, Mordant Orange, Reactive Black, Direct Yellow, Reactive Orange, Remazol, Wantex Red and Wantex Yellow dye, successfully removing up to 68.35% and 79.50% Methylene Blue and Basic Fuchsine in a medium containing 3 mM CuSO_4_, respectively. Further investigations are required to analyse the genetic composition of multi-resistant bacteria to optimise the effectiveness of indigenous bacterial isolates as bioremediation agents.

**Highlights**

- Indigenous bacterial strain *Acinetobacter* sp. IrC1 resisted up to 8 mM of CuSO_4_ and accumulated up to 292.93 mg/g dry weight of copper cells based on spectrophotometric analysis.
- IrC1 isolates resisted up to 500 ppm 10 types of textile dye, namely Methylene Blue, Malachite Green, Congo Red, Mordant Orange, Reactive Black, Direct Yellow, Reactive Orange, Remazol, Wantex Red and Wantex Yellow dye.
- *Acinetobacter* sp. IrC1 is a multi-resistant strain that performs copper resistance and dye decolourisation simultaneously, successfully removing up to 68.35% of Methylene Blue and 79.50% of Basic Fuchsine dye in a medium containing 3 mM CuSO_4_.

## INTRODUCTION

Heavy metal contamination is a major environmental issue, a threat to ecosystem sustainability and human health that shows an alarming rate due to the ever-increasing anthropogenic activities. Heavy metals such as copper are commonly defined as metals with a density of over 5 g/cm^3^ (Tutic *et al*. 2015) and required in low concentrations (nM) for biochemical and physiochemical processes, including cell metabolism. However, heavy metals are toxic to both micro- and macro-organisms at higher concentrations (μM to mM) (Singh *et al*. 2011; Tchounwou *et al*. 2012). Heavy metal deposition disrupts the atmospheric biogeochemical cycle, negatively altering environmental components (Ali *et al*. 2019). Heavy metals can reach humans via multiple food chain networks due to their non-biodegradable (Nkwunonwo *et al*. 2020) and easily transferable properties between biota at different trophic levels (Ali & Khan 2019). Plants and animals—especially those involved in a marine food web—may transfer heavy metals to each other. It causes bioaccumulation and eventual biomagnification once it reaches the higher trophic levels (Miller *et al*. 2020). Heavy metals that require immediate concern include arsenic, cadmium, chromium, copper, lead, nickel, mercury and zinc (Wuana & Okieimen 2011).

Copper has been noted as one of the most toxic pollutants in the marine environment (Okocha & Adedeji 2012). Furthermore, the demand for copper is expected to increase by 50% in the next two decades due to its extensive use in construction, electronics, electroplating, transportation and steel industries that are rapidly growing because of population growth, urban expansion and industrial development. Other anthropogenic activities that lead to the production and discharge of excess copper include agriculture, petroleum refining, mining, and metallurgy (Shrivatava 2009). Consequently, all levels of the food chain will be exposed to elevated copper concentration rates, significantly reducing biodiversity and impacting human health. Copper ingestion via the food chain may cause adverse physiological and neurological health effects as copper mostly targets the liver and brain (Gaetke *et al*. 2014). Copper toxicity mostly targets the liver, breaking down liver cells and releasing large amounts of copper into the blood circulation. Damaged blood constituents often lead to organ-function abnormalities or vital organ damage that may occur in the lungs, liver and kidney, causing acute failure and, thereby, death (National Research Council (US) Committee on Copper in Drinking Water 2000). Copper toxicity may also damage central nervous systems, leading to neurodegenerative disorders (Giampietro *et al*. 2018).

Synthetic dye contamination is also recognised but underestimated as a major pollution issue. Dye contamination jeopardizes the quality of aquatic environmental components by exerting toxic effects on plants, animals and humans. Large-scale production and widespread application of dyes in sectors such as textile, tannery, cosmetics, food, paper and printing, pharmaceutics, and electroplating pose a high demand for various synthetic dyes such as Methylene Blue, Malachite Green, Congo Red, Mordant Orange, Reactive Black, Direct Yellow, Basic Fuchsine, Reactive Orange, Disperse Orange, Remazol, Wantex Red and Wantex Yellow (Drumond *et al*. 2013; Tkaczyk *et al*. 2020). Synthetic dyes are chemically diverse colourants extensively used in textile industries despite their toxicological properties. These colourants are equipped with high affinity, a property that allows them to easily bind with cellulosic fiber (Karim *et al*. 2018). However, the textile dyeing process generates a large volume of post-production wastewater containing an average of 15%–50% or at least 5% of the textile dyes (Tkaczyk *et al*. 2020; Lellis *et al*. 2019). Dyes are synthesised industrially and highly versatile, stable and long-lasting colourants in natural environments. Therefore, aquatic ecosystems consider them micropollutants (ng/L to μg/L) (Stasinakis & Gatidou 2016). When released into water resources, dyes will break down into recalcitrant by-products that exert toxic and mutagenic effects on living aquatic organisms, such as inhibiting growth due to a disrupted photosynthesis process and dissolved oxygen levels. Meanwhile, the azo group in dyes exert mutagenic and carcinogenic effects as they are converted to aromatic amines, which act as potential human carcinogens when incorporated into the food chain and crossed between different trophic levels (Abe *et al*. 2018). Consequently, humans atop the highest trophic level in food chains carry a high risk of contamination due to biomagnification. The toxic properties of synthetic dye by-products include allergic, carcinogenic, and mutagenic effects. Specifications include allergic dermatitis, skin irritation, liver and bladder cancer, and even central nervous system disorders (Khan & Malik 2018).

Conventional treatment methods for removing heavy metals from water resources involve physiochemical processes such as chemical precipitation, membrane filtration, flocculation, electrolysis and crystallisation, evaporation recovery, ion exchange, reverse osmosis, oxidation/reduction. These processes show limitations such as high costs when treating large bodies of water, ineffectiveness at low concentrations of metal (less than 10 mg/L), and the generation of large quantities of sludge and other toxic products that require careful disposal (Pertile *et al*. 2020). Thus, conventional methods are considered inefficient, ineffective, and uneconomical to remove heavy metal contaminants from the aquatic environment. On the other hand, synthetic dyes are difficult to remove via conventional methods as they are stable to light and oxidising agents, such as hydrogen peroxide and potassium dichromate, resistant to aerobic digestion (Mahbub *et al*. 2012). Therefore, it is important to develop a novel approach of environmentally friendly, efficient, and economical alternative methods to remove these two major contaminants from large bodies of water immediately.

Bacteria-based bioremediation, a natural and cost-effective process utilising bacterial isolates, offers a high potential to restore the quality of the copper and dye-contaminated water through bioaccumulation and decolourisation. Copper and synthetic dye are both non-biodegradable and toxic to microbes. Thus, several microorganisms that reside in contaminated sites have developed a resistance mechanism to counter toxicity and continue thriving, explaining why they record higher survival and adaptation rates in any given environment (Igiri *et al*. 2018). Microorganisms that survive well despite being exposed to high concentrations of copper could be used as bioremediation agents. They can conduct copper bioaccumulation, in which excess copper is accumulated within the membrane fraction and inside the cytoplasm (Irawati *et al*. 2012). Furthermore, microorganisms that survive in dye-contaminated marine sites may also be bioremediation agents. They can degrade or even decolourise dyes through an enzymatic activity that alters the chemical structures of the dye. Meanwhile, the dye bioremediation process mostly depends on abiotic factors such as aeration, pH, and temperature, along with other factors such as dye concentration, co-substrate presence, dye-degrading enzymatic activity (Ogugbue & Sawidis 2011).

Textile manufacturing factories consistently discharge waste containing excess dangerous metals such as copper, lead, nickel, cadmium and chromium into their surroundings (Bhardwaj *et al*. 2014). Thus, copper and dye are highly likely to be simultaneously found among industrial waste in the environment. Previous studies show that copper-resistant bacteria isolated from polluted bodies of water can be used as copper bioremediation agents. Genera *Cupriavidus, Acinetobacter* (Irawati *et al*. 2012)*. Bacillus altitudinis MT422188* (Khan e*t al*. 2022), *Klebsiella pneumoniae MB375, Staphylococcus* sp. *MB377, Klebsiella oxytoca MB381* (Tahir *et al*. 2021), *Pseudomonas veronii* (Busnelli & Vullo *2022*), *Citrobacteria freundii* (Sharma & Fulekar 2009) have been reported as copper resistant bacteria employed to reduce copper concentrations or bio-transform the chemical properties of copper into less-toxic forms. However, the study on multi-resistant bacteria to copper and dyes has so far been quite limited.

*Acinetobacter* sp. IrC1 are copper-resistant bacteria isolated from activated sludge from a wastewater treatment plant in Rungkut-Surabaya, Indonesia (Irawati *et al*. 2012). Ghodake *et al*. (2011) demonstrated the resistance of *Acinetobacter calcoaceticus* to 20 different synthetic dyes, including Methylene Blue, Malachite Green and Congo Red. Given the impact of copper and dye pollution on ecological and human health, it is significant to increase waste treatment efficiency by exploring the multi-potency of *Acinetobacter* sp. IrC1 against copper and dyes. Therefore, this study aims to:

1. Determine the effect of various copper concentrations on bacterial growth.
2. Measure the ability of bacteria to accumulate copper.
3. Observe bacterial growth on 12 types of dye frequently used in the textile industry, i.e., Methylene Blue, Malachite Green, Congo Red, Mordant Orange, Reactive Black, Direct Yellow, Basic Fuchsine, Reactive Orange, Disperse Orange, Remazol, Wantex Red and Wantex Yellow dye.
4. Determining the ability of this to decolourise selected dyes.

## MATERIALS AND METHODS

### Bacterial Culture and Growth Medium Preparation

*Acinetobacter* sp. IrC1 is a copper-resistant bacterial strain with accession number JX009133. *Acinetobacter* sp. IrC1 was Gram negative bacterium isolated from activated sludge discharged from a sewage treatment plant in Surabaya, Indonesia.

Based on genes 16S rDNA 98.41% analysis, *Acinetobacter* sp. IrC1 had genes similarity with *Acinetobacter calcialiticus* of 98.41%. (Irawati *et al*. 2012). Luria Bertani Miller (LB) was used as the growth medium with the following composition per liter: tryptone 10, yeast extract 5, and sodium chloride 10 g. A solid medium was prepared by dissolving 25 g of Luria Bertani broth and 25 g of American Bacteriological Agar into one liter of dH_2_O. *Acinetobacter* sp. IrC1 starter culture was prepared by inoculating bacterial isolates from solid LB medium into 5 mL of liquid LB medium, followed by incubating at 37°C until it reached a logarithmic phase.

### Bacterial Copper-Resistance and Copper Accumulation Analysis Method

Copper stock with a concentration of 1000 mM CuSO_4_ was prepared and sterilised by membrane filter to supplement solid and liquid LB medium with appropriate copper concentrations (4 mM, 5 mM, 6 mM, 7 mM, 8 mM and 9 mM). Approximately 250 μL of the starter culture was inoculated into 25 mL of liquid LB medium containing various CuSO_4_ concentrations, incubated at 37°C and shaken at 200 rpm. Copper resistance was observed through cell turbidity measurement (optical density) using a Labo Med spectrophotometer at 600 nm.

Bacterial cultures were harvested using centrifugation at 5000 xg for 20 min to collect cells. The cells were were freeze-dried to obtain the dry weight of the cells. The dry weight of the cell was dissolved with 20 mL dH_2_O then destructed by heating at 110°C and adding HNO_3_ to break the Cu bonds from the organic matter present in the medium. Destruction was carried out until the sample became clear. The concentration of Cu in the cell dry weight samples was analysed using an Atomic Absorption Spectrophotometer to obtain Cu bioaccumulation values (Cha & Cooksey 1991).

### Bacterial Dye-Resistance and Decolourisation Analysis Method

#### Bacterial dye-resistance3

Dye stock with a concentration of 10,000 ppm was formulated by dissolving 1 g of Methylene Blue, Malachite Green, Congo Red, Mordant Orange, Reactive Black, Direct Yellow, Basic Fuchsine, Reactive Orange, Disperse Orange, Remazol, Wantex Red and Wantex Yellow dye each into 100 mL of sterile distilled water followed by membrance sterilisation. Solid LB media were supplemented with 200 ppm and 500 ppm of each dye. A loopful of *Acinetobacter* sp. IrC1 was streaked onto each dye-supplemented media using the four quadrants method. Bacterial plating was performed in triplicates to maintain consistency and then were incubated at 37°C for 48 h. Decolourisation ability can be observed from formation of clear zone around the colony. The same treatment were carried out on solid medium containing each dye supplemented with of 3 mM or 5 mM CuSO_4_ to determine the effect of CuSO_4_ towards bacterial growth and clear zone formation.

#### Decolourisation analysis

A starter culture was produced by transferring one inoculation loop’s of *Acinetobacter* sp. IrC1 from test tubes of slanted growth agars to 50 mL of liquid LB medium, then was incubated in an incubator shaker with a speed of 150 rpm and a temperature of 37**°**C. Starter culture growth was measured using a spectrophotometer, measured at wavelength 600 nm. The starter culture was considered fit to be used once the optical density (OD) of the culture reaches 0.6. About 1% of each starter culture was inoculated to 10 mL of liquid LB medium, each containing 12 different dyes with concentrations of 200 ppm or 500 ppm. The same procedures and conditions were used to inoculate the culture to 10 mL liquid LB containing a dye and 3 mM CuSO_4_. Bacterial isolates were incubated for 24 h in an incubator shaker with a speed of 150 rpm and a temperature of 37**°**C. After 24 h and 48 h, as much as 1,000 uL of each liquid culture were then transferred to sterile microtubes and centrifuged for one minute at 15,000 rpm. The supernatant formed was analysed using a spectrophotometer at 300 nm–900 nm. The medium containing no dye was used as a control (Irawati *et al*. 2022). The percentage decolourisation value was measure using the following formula (John *et al*. 2020):


$$\%\;\text{of decolourisation}=\frac{\text{Absorbance of control}-\text{Absorbance of treated sample}}{\text{Absorbance of control}}\times 100\%$$

## RESULTS

### Copper-Resistance

**Figure 1:**
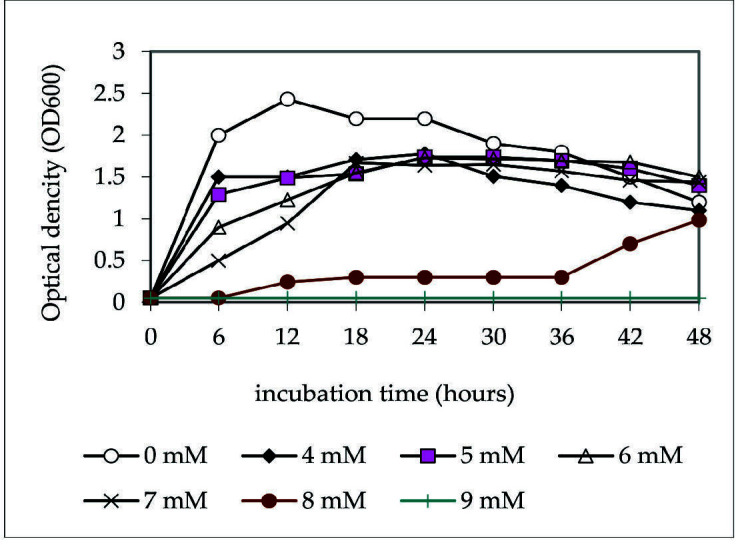
The effect of copper concentration on bacterial growth.

Fig. 1 shows the comparison of *Acinetobacter* sp. IrC1 growth on medium without copper to enriched media supplemented with 4 mM, 6 mM, 7 mM, 8 mM and 9 mM of CuSO_4_. IrC1 immediately entered a logarithmic phase when grown on both mediums without Cu and enriched media (4 mM to 7 mM). Despite the absence of an “adaptation,” otherwise known as the lag phase when exposed to different levels of CuSO_4_, IrC1 survived well, albeit at a slower growth rate. However, differences in the speed bacterial growth rate on enriched media were observed. The higher the CuSO_4_ concentration, the slower the bacterial growth. Growth of *Acinetobacter* sp. IrC1 on 4 mM and 5 mM medium showed a higher rate than on 6 mM and 7 mM. Fig. 1 demonstrates that adding 8 mM CuSO_4_ led to a 6-hour lag phase, while the absence of bacterial growth was observed on 9 mM-supplemented with the medium. This observation indicates that 8 mM is the threshold concentration for copper to exert growth-hindering toxicity on bacterial cells.

### Copper Accumulation

**Figure 2:**
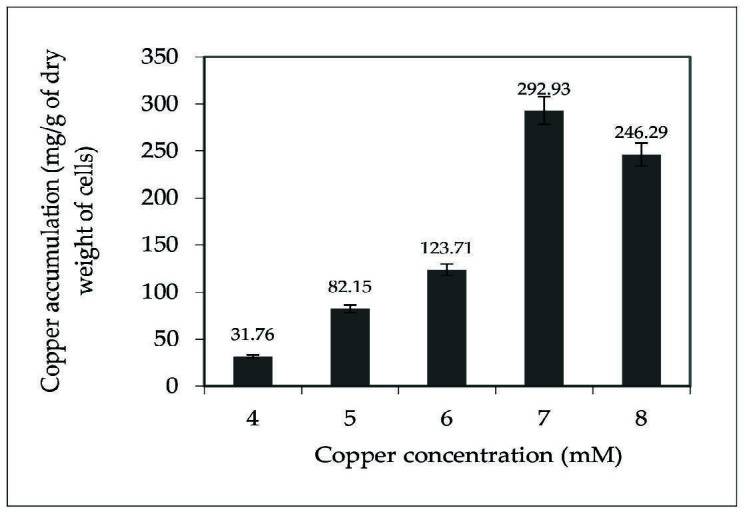
Copper accumulation by *Acinetobacter* sp. IrC1 at various copper concentrations.

Copper accumulation analysis of *Acinetobacter* sp. IrC1 in media containing 4 mM to 8 mM of CuSO_4_ (Fig. 2) shows that *Acinetobacter* sp. IrC1 can accumulate copper intracellularly, presumably one of its resistance mechanisms conducted to tolerate copper stress. At 4 mM, IrC1 marked its lowest copper accumulation rate at 31.76 mg/g dry weight of cells. Copper accumulation in the cells increased with increasing of CuSO_4_ concentration, reaching its peak at 7 mM before decreasing at 8 mM. This decrease supports the concept that 8 mM is the threshold concentration for copper toxicity. The highest concentration of copper accumulation was observed at 292.93 mg/g of cell dry weight when grown on media supplemented with 7 mM of CuSO_4_.

### Dye-Resistance

**Figure 3:**
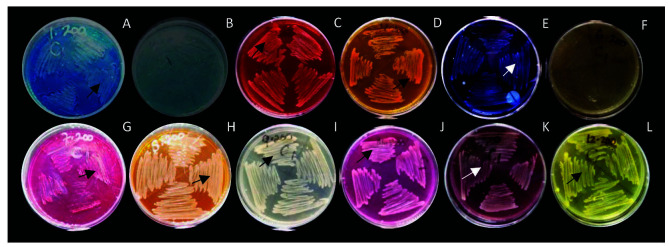
Growth of *Acinetobacter* sp. IrC1 on solid LB medium supplemented with 200 ppm various dyes. (A) Methylene Blue, (B) Malachite Green, (C) Congo Red, (D) Mordant Orange, (E) Reactive Black, (F) Direct Yellow, (G) Basic Fuchsine, (H) Reactive Orange, (I) Disperse Orange, (J) Remazol, (K) Wantex Red, (L) Wantex Yellow. Arrow represents bacterial colonisation.

The growth analysis presented in Fig. 3 reports the growth of *Acinetobacter* sp. IrC1 on enriched media containing 12 dyes: Methylene Blue, Malachite Green, Congo Red, Mordant Orange, Reactive Black, Direct Yellow, Basic Fuchsine, Reactive Orange, Disperse Orange, Remazol, Wantex Red and Wantex Yellow. *Acinetobacter* sp. IrC1 was successfully cultivated on 10 dyes, excluding Malachite Green and Direct Yellow, when not supplemented with CuSO_4_.

On the contrary, Table 1 showed that 3 mM CuSO_4_ inhibited bacterial growth on 200 ppm and 500 ppm dye-supplemented media. Adding CuSO_4_ confined bacteria to only grow on 200 ppm Methylene Blue and Basic Fuchsine medium, and on 500 ppm Methylene Blue medium. An identical phenomenon was performed by *Acinetobacter* sp. CN5, as decreased bacterial growth in media containing Methylene Blue, Congo Red, Basic Fuchsine and Wantex Red appeared when the concentration of CuSO_4_ increased from 3 mM to 5 mM (Alam *et al*. 2009).

### Decolourisation

**Figure 4:**
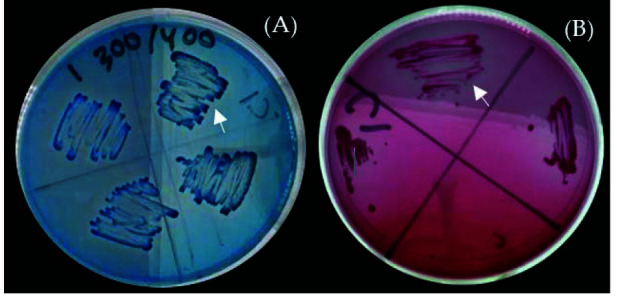
Decolourisation activity of *Acinetobacter* sp. IrC1 on solid LB medium supplemented with 3 mM CuSO_4_ + 200 ppm dyes. (A) Methylene Blue, (B) Basic Fuchsine. Arrows represent bacterial colonisation. White arrows show clear zone area

Fig. 4 shows that adding 3 mM CuSO_4_ to 200 ppm Methylene Blue and 200 ppm Basic Fuchsine led to decolourisation, as evidenced by the clear zone formed around the bacterial colonies. The same phenomenon also occurred in *Acinetobacter* sp. CN5 as observed by the clear zone around bacterial colonies growing on a medium containing Methylene Blue and Basic Fuchsine (Alam *et al*. 2009). This confirms that the genus *Acinetobacter* has the potential to grow on, as well as decolourise methylene blu and basic fuchsine dye.

**Figure 5:**
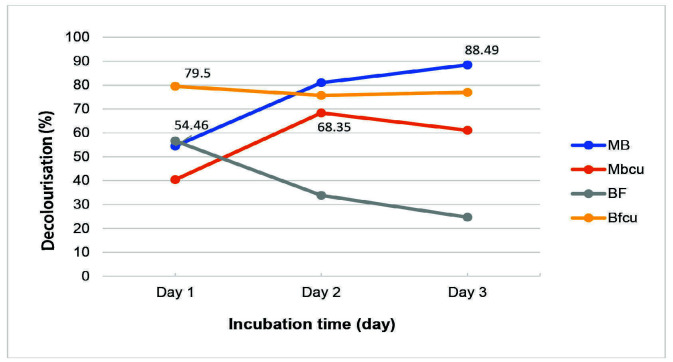
The influence of copper added to decolourisation ability of *Acinetobacter* sp. IrC1. (MB) 200 ppm Methylene Blue, (Mbcu) 200 ppm Methylene Blue plus 3 mM CuSO_4_, (BF) 200 ppm Basic Fuchsine, (BFcu) 200 ppm Basic Fuchsine plus 3 mM CuSO_4_.

Decolourisation ability of *Acinetobacter* sp. IrC1 on Methylene Blue and Basic Fuchsine were 88.49% and 56.63%, as calculated on the third and first day of observation (Fig. 5). Adding CuSO_4_ to Methylene Blue medium decreased the decolourisation ability of bacteria from 88.49% to 68.35%, but the ability of bacteria to decolourise Basic Fuchsine increased from 68.35% to 79.50%. This inverse relation shows that copper (II) ions play a significant role in optimising the decolourisation process of Basic Fuchsine.

## DISCUSSION

### Copper-Resistance

Results of the copper-resistance test suggest that *Acinetobacter* sp. IrC1 has a minimum inhibitory concentration of 9 mM CuSO_4_. Samanovic *et al*. (2012) reported that copper acts as a micronutrient source in various cellular processes to support bacterial growth and development at low concentrations. Argüello *et al*. (2013) support this concept by stating that copper functions as a co-factor in redox reactions, electron transport, an intracellular oxidative respiration. It can thus be inferred that 8 mM is the threshold concentration for copper to transition from supporting to hindering to the growth of bacterial cells.

Interestingly, *Acinetobacter* sp. IrC1 was able to adapt to copper toxicity by developing a resistance mechanism upon entering a lag phase. Bertrand (2019) termed this phenomenon “tolerance by lag” as lag phases allow the adaptation required to equip bacteria with all the biochemical properties necessary to maintain their survival, growth and development despite being exposed to copper toxicity. When bacterial cells are exposed to toxic levels of contaminants such as copper, they experience a decrease in the number of viable cells and require more time for physiological recuperation (Dupont & Augustin 2009). Furthermore, lag phases allow the bacteria to induce DNA repair mechanisms of the damaged cellular components before exiting the lag phase and undergoing exponential growth (Larsen *et al*. 2006). It is also important to note that, at 8 mM Cu concentration, *Acinetobacter* sp. IrC1 underwent a second lag phase from hours 12 to 36. A second lag phase allowed *Acinetobacter* sp. IrC1 to produce secondary metabolites in efforts to further equip themselves with the necessary properties needed to survive and thrive. The occurrence of the second lag phase suggests that, during the first lag phase, *Acinetobacter* sp. IrC1 initiated a novel transcriptional program to synthesise the components required for cellular multiplication during the exponential phase. Meanwhile, the second phase occurred due to the necessary synthesis of cellular components required to reparate macromolecular damage sustained during the stationary phase (Bertrand 2019; Rolfe *et al*. 2012).

*Acinetobacter* sp. IrC1 has been successfully proven to be a highly copper-resistant bacterium. The bacterium showed a level of copper resistance similar to the resistance levels of indigenous bacteria isolated from several copper-contaminated marine sites in Indonesia. These locations include Kemisan River, Tangerang, where the indigenous bacteria isolated were able to grow on media containing 3 to 10 mM of CuSO_4_ (Irawati *et al*. 2017), and Sukolilo River in East Java since the bacteria were able to grow on 3 to 9 mM of CuSO_4_, as well as Cikapundung and Cisadane River in West Java where the bacteria grew on mediums supplemented with up to 6 to 9 mM of CuSO_4_ (Nurlaila *et al*. 2021). Based on these findings, IrC1 may be considered a potential bioremediation agent for copper-contaminated sites, especially in Indonesia, where the issue of copper contamination continues to prevail and grow at an alarming rate.

### Copper Accumulation

The highest concentration of copper accumulation was 292.93 mg/g of dry cell weight on media supplemented with 7 mM of CuSO_4_. The bacterial cell membrane likely lacks the availability and capacity of binding sites to accumulate copper at toxic levels such as 8 mM due to oversaturation or extended overexposure (Irawati *et al*. 2017). Copper bioaccumulation ability of *Acinetobacter* sp. IrC1 was higher than *Acinetobacter sp*. IrC2 accumulated up to 138.96 mg/g dry weight of copper cells (Irawati, Kusumawati, et al. 2015), but lower than *Cupriavidus* sp. IrC4 accumulated up to 367.78 mg/g dry weight of copper cells (Irawati, Yuwono, et al. 2015). All three species were isolated from activated sludge in an industrial wastewater treatment plant in Rungkut-Surabaya, Indonesia. Bioaccumulation is an active process involving metabolism in which bacterial proteins uptake and sequester metal ions, then transport them across the cell membrane to be stored in the intracellular space for future utilisation (Timková *et al*. 2018).

Bioaccumulation of heavy metals is highly dependent on the intrinsic structure, biochemical properties, genetic and physiological adaptations of the microorganism, and the availability and toxicity of metals in its surroundings. According to Rehman *et al*. (2008), intracellular copper uptake is performed through various biochemical mechanisms involving the synthesis of adsorptive agents, especially proteins, during their lag phase. These agents are responsible for binding copper ions onto the cell surface, where they wait to be actively transported across the cell membrane and into the cytoplasm or periplasm, where it is accumulated (Timková *et al*. 2018).

The mechanism of bacterial resistance to copper in *Pseudomonas syringae* is encoded by the CopABCD operon gene which produces CopA, CopB, CopC and CopD proteins. Cu ions enter the cell through the CopB protein in the outer membrane. Cu ions are mostly bound by CopA and CopC proteins in the periplasm to avoid excessive entry of Cu ions into the cytoplasm. Excess Cu ions in the cytoplasm cause oxidative stress so that copper-resistant bacteria will remove Cu ions outside the cell through the help of CopB protein (Cha & Cooksey 1991). This mechanism involved enzymes that play a role in the detoxification process, namely, superoxide dismutase (SOD) (Kong *et al*. 2020), multicopper oxidase (MCO) (Wen *et al*. 2014), and Universal stress protein (USP) (Chi *et al*. 2019) to avoid cell damage under copper stress.

In the lag phase, bacteria will synthesize SOD as a metalloenzyme which acts as an antioxidant and is responsible for protecting DNA from damage (Kong *et al*. 2020). MCO is an enzyme that contains four copper units and undergoes increased synthesis when bacteria are grown in copper-containing media (Wen *et al*. 2014). MCO plays a role in the mechanism of Cu resistance and detoxification in the periplasm by oxidising toxic Cu^+^ into ions to Cu^2+^ (Alquethamy *et al*. 2019). USP plays a role in preventing denaturation of macromolecules and repairing and protecting nucleic acids when bacteria experience copper oxidative stress (Chi *et al*. 2019).

### Dye-Resistance

*Acinetobacter* sp. IrC1 was incapable of growing on Malachite Green and Direct Yellow despite the absence of CuSO_4_. The results of previous studies reported that Malachite Green and Direct Yellow are toxic because they are antibacterial. These results are plausible as (Junqueira *et al*. 2010) exhibited the dynamic antimicrobial properties of Malachite Green against a total of 36 *Candida*, *Enterobacteriaceae* and *Staphylococcus* strains. Mehrizi *et al*. (2009) also reported the antimicrobial effects of direct yellow treated fabric combined with metal salts as a mordant against *S. aureus* and *E. coli* as a result of ionic sulfate group and copper (II) ion coordination.

*Acinetobacter* sp. IrC1 was successfully cultivated in media containing various dyes due to the development of an enzyme-mediated resistance mechanism. Hsueh *et al*. (2017) state that bacteria isolated from a contaminated site can adapt and modify their biochemical and physiological properties to conduct a catabolic activity, such as the degradation of synthetic dyes. Such a phenomenon occurs as bacteria develop a resistance mechanism to protect its cells against dye toxicity, especially in prolonged exposure. Balapure *et al*. (2014) and Jayapal *et al*. (2018) suggest that the biodegradation of dyes, specifically those with aromatic amine structures, occurs through two main phases. Azo bound are first broken down through the liberation of aromatic amine structures, and aromatic compound mineralisation then follows. These corroborations align with Jadhav *et al*. (2016). They suggested that bacteria may secrete extracellular reductive enzymes, including azo reductase or oxidative enzymes such as hydrogenase, laccase and peroxidase to help degrade dyes under stressful conditions. According to Jamee and Siddique (2019), bacteria mineralise complex and toxic dyes into simpler compounds with the help of oxidative and reductive enzymes. Then, successfully mineralised dye compounds will be further degraded to be utilised as an energy source for future metabolic processes.

Copper toxicity, interaction and coordination with the chemical compounds found in dye compositions are the factors that affect bacterial dye resistance. Mehrizi *et al*. (2009) identified sulfonate and azo groups in dyes as the main drivers that interact with copper (II) ions to promote antimicrobial activity, as evidenced by up to 100% reduced *S. aureus* and *E. coli* growth on direct yellow 12, direct red 23, direct red 31, and direct black 38 dye. Chaturvedi and Verma (2015) demonstrated that CuSO_4_ toxicity to bacterial cells also affected dye-resistance as observed through reduced malachite green decolourisation activity by *Ochrobactrum pseudogrignonense* strain CGUPV1 in the presence of CuSO_4_.

### Decolourisation

The success of *Acinetobacter* sp. IrC1 in decolourisation methylene blue and basic fuchsine dye in the presence of copper is attributable to its intrinsic nature. Al-Sulami and Jaafar (2015) explained that physiological and genetic adaptations of bacteria allow certain species to accumulate heavy metals and remove dye colours simultaneously. Decolourisation can be defined as the process of removing dyes from stained specimens (Saratale *et al*. 2011).

Bacteria such as *Acinetobacter* sp. that harbor azo-reductases, laccases and peroxidases are capable of decolourising dyes by altering specific enzymatic activities involved (Khandare & Govinwar 2015; Pinheiro *et al*. 2022). According to Alam *et al*. (2009) and Duran-Rivera *et al*. (2018), bacteria were able to remove Methylene Blue using lignin peroxidase (LiP) and laccase. Kang *et al*. (2014) also state that bacteria can decolourise basic fuchsine with the assistance of LiP. LiP activates the process of decolourising both dyes by cleaving the aromatic ring structure, whereas laccase cleaves the -N(CH_3_)N functional group from the chemical structure of Methylene Blue dye. Based on previous research by Chen *et al*. (2002), copper (II) ions increase the enzymatic activity of cationic peroxidases, optimising the lignification required for decolourisation. Furthermore, laccases can benefit from the presence of copper (II) ions as redox mediators, significantly improving decolourisation rates. Thus, it is suggested that copper addition helps create favourable conditions for bacteria to perform decolourisation of basic fuchsine (Pinheiro *et al*. 2022).

Decolourisation is a process highly dependent on the Fenton reaction cycle as hydroxyl radicals’ rapid production and effective longevity help improve decolourisation efficiency (Goodell *et al*. 2004). Copper ion surplus catalyzes a Fenton-like reaction that generates an abundant supply of hydroxyl radicals (Kim *et al*. 2018). Jadhav *et al*. (2012) specifically reported oxidative stress generation and SOD induction followed by decolourisation of an azo dye after bacterial treatment. They provided a proof of concept that the supplementation of copper into dye plays a significant role in optimising decolourisation.

*Acinetobacter* sp. IrC1. (88.49%) demonstrated a relatively high Methylene Blue decolourisation ability compared to *Pseudomonas aeruginosa* (21.31%), *Acinetobacter* sp. CN5 (57.64%), *R. mannolytica* (60.3%), and *C. Aquatica* (67.9%), albeit lower than *Desmodesmus* sp. (98.6%) (Irawati *et al*. 2022; Al-Fawwaz & Abdullah 2016; Michelle *et al*. 2020). Decolourisation of Basic Fuchsine by *Acinetobacter* sp. IrC1 (79.50%) also produced results higher than *Saccharomyces ceresvisiae* isolated from salt water (60.39%) and pine wine (72.61%), but lower than *Acinetobacter* sp. CN5 (91.37%) and *Aeromonas hydrophila* (93%) (Ogugbue & Sawidis 2011; Irawati *et al*. 2022; Shnada *et al*. 2015). Based on these results, *Acinetobacter* sp IrC1 carries the potential to be utilised as decolourising agent for Methylene Blue and Basic Fuchsine.

To date, research on copper and dye multi-resistant bacteria is still limited, while the potential of local marine microbes remains largely unexplored. For instance, Ghodake *et al*. (2011) reported that *Acinetobacter calcoaceticus* was able to decolourise 20 different textile dyes from 8 different classes, namely azo, reactive azo, disperse, triphenylmethane (basic fuchsine), direct, thiazin, pthalocyanin and heterocyclic (methylene blue) dye, but not resistant to copper (Ghodake *et al*. 2011). Dianrevy (2017) reported that the genus *Bacillus, Pseudomonas* and *Zooglea* isolated from batik textile wastewater in Yogyakarta, Indonesia, removed 74.63% copper and resisted napthol red dye. In contrast,Taştan *et al*. (2010) reported that the indigenous fungus *Aspergillus versicolor* isolated from Batman, Turkey, can accumulate 16.28% copper and at least 94% of 150 mg/L remazol blue dye.

## CONCLUSION

The results of this work demonstrated that *Acinetobacter* sp. IrC1 is a copper and dyes-multi-resistant bacterium. *Acinetobacter* sp. IrC1 exhibited resistance to 8 mM CuSO_4_ and accumulated up to 292.93 mg/g of cells dry weight of copper. *Acinetobacter* sp. IrC1 was also resistant to 500 ppm of 10 different dyes, including Methylene Blue, Malachite Green, Congo Red, Mordant Orange, Reactive Black, Direct Yellow, Reactive Orange, Remazol, Wantex Red and Wantex Yellow. *Acinetobacter* sp. IrC1 was found capable of decolourising Methylene Blue and Basic Fuchsine up to 68.35% and 79.50% in a medium containing 3 mM CuSO_4_, respectively. It is, therefore, of importance to explore *Acinetobacter* sp. IrC1 is further an eco-friendly, cost-efficient, and effective bioremediation agent for future manufacturing or discharging processes. The exploration of dye and copper multi-resistance bacteria would enhance the possibilities of applying bacteria in solving the problem of water pollution due to copper and dyes.

## Figures and Tables

**Table 1:** Growth of *Acinetobacter* sp. IrC1 on various concentrations of copper and dye.

Treatment	Growth of *Acinetobacter* sp. IrC1											

	1	2	3	4	5	6	7	8	9	10	11	12
200 ppm dye	+	−	+	+	+	−	+	+	+	+	+	+
500 ppm dye	+	−	+	+	+	−	+	+	+	+	+	+
200 ppm dye + 3 mM CuSO_4_	+	−	−	−	−	−	+	−	−	−	−	−
500 ppm dye + 3 mM CuSO_4_	−	−	−	−	−	−	+	−	−	−	−	−

*Note*: (1) Methylene Blue, (2) Malachite Green, (3) Congo Red, (4) Mordant Orange, (5) Reactive Black, (6) Direct Yellow, (7) Basic Fuchsine, (8) Reactive Orange, (9) Disperse Orange, (10) Remazol,(11) Wantex Red, (12) Wantex Yellow.
